# Long-Term Results of Combined Tunica Albuginea Plication and Penile Prosthesis Implantation for Severe Penile Curvature and Erectile Dysfunction

**DOI:** 10.1155/2014/818623

**Published:** 2014-03-25

**Authors:** Luigi Cormio, Paolo Massenio, Giuseppe Di Fino, Giuseppe Lucarelli, Vito Mancini, Giuseppe Liuzzi, Giuseppe Carrieri

**Affiliations:** Department of Urology and Renal Transplantation, University of Foggia, Viale L. Pinto 1, 71121 Foggia, Italy

## Abstract

Penile prosthesis implantation is the recommended treatment in patients with penile curvature and severe erectile dysfunction (ED) not responding to pharmacotherapy. Most patients with mild-to-moderate curvature can expect cylinder insertion to correct both ED and penile curvature. In patients with severe curvature and in those with persistent curvature after corporeal dilation and prosthesis placement, intraoperative penile “modelling” over the inflated prosthesis has been introduced as an effective treatment. We report for the first time the long-term results of a patient treated with combined penile plication and placement of an inflatable penile prosthesis.

## 1. Introduction

Penile prosthesis implantation is the recommended treatment in patients with penile curvature and severe erectile dysfunction (ED) not responding to pharmacotherapy [1]. Most patients with mild-to-moderate curvature can expect cylinder insertion to correct both ED and penile curvature. In patients with severe curvature and in those with persistent curvature after corporeal dilation and prosthesis placement, intraoperative penile “modelling” over the inflated prosthesis has been introduced as an effective treatment [2–4]. However, this procedure is not effective in all cases and involves a 4% risk of urethral laceration [3]. Tunical incisions, with or without grafting, have been used to correct residual curvatures after penile modelling; however, they involve increased morbidity, particularly in terms of hematoma and infection [5–7].

In 2004, Rahman et al. [8] described a simplified technique for correcting severe penile curvature and erectile dysfunction using combined penile plication and placement of an inflatable penile prosthesis. Herein, we report for the first time the long-term results of a patient we treated with this procedure.

## 2. Case Report

A 60-year-old Caucasian man underwent bilateral nerve-sparing open retropubic radical prostatectomy because of a pT1c Gleason 7 prostate cancer. His preoperative International Index of Erectile Function-5 (IIEF-5) score was 21. Both operative course and postoperative course were uneventful. Final pathology revealed a pT2cN0 prostate cancer Gleason 7 with three positive surgical margins. He therefore received adjuvant radiotherapy. Following such treatments, he noticed a progressive deterioration of his erectile function, despite the use of phosphodiasterase-5 (PDE-5) inhibitors and intracavernous injection (ICI) with prostaglandin E1 (PGE1), with progressive dorsal penile curvature. At three-year follow-up, his prostate specific antigen (PSA) was 0.001 ng/mL; he was fully continent but unhappy with his sexual life. Specifically, he reported self-ICI of 20 mcg PGE1 to result into penile tumescence not sufficient for intercourse with severe dorsal penile curvature; his IIEF-5 score with self-ICI of 20 mcg PGE1 was 10. Penile colour duplex ultrasound findings, after ICI of 40 mcg PGE1 [9], were consistent with severe veno-occlusive dysfunction (resistance index 0.78); there also was relevant dorsal penile curvature, which however could not be adequately measured as the patient obtained only penile tumescence and not rigidity. Following extensive discussion about the available surgical options for the correction of erectile dysfunction and severe penile curvature, he was scheduled for penile prosthesis implantation with tunica albuginea plication.

**Figure 1:**
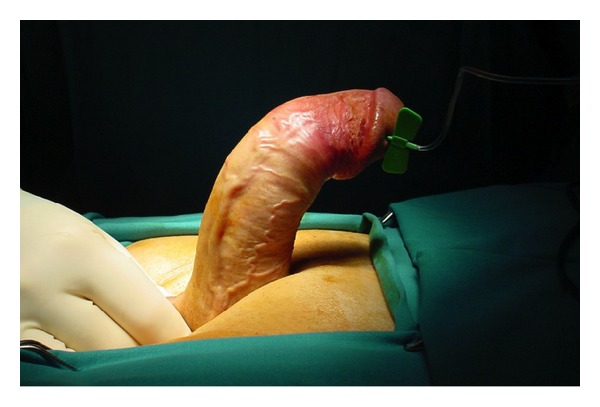
Artificial erection (intracavernous injection of saline and occlusion at the penile base with finger pressure) showing approximately 70° curvature of dorsal penile shaft.

**Figure 2:**
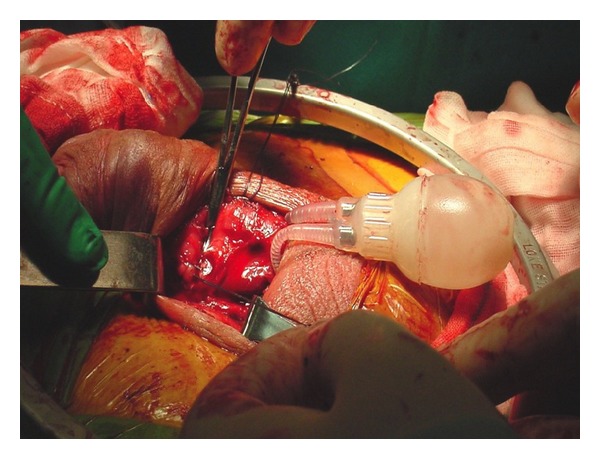
The first row of plication sutures, placed before insertion of the penile prosthesis, is tied.

**Figure 3:**
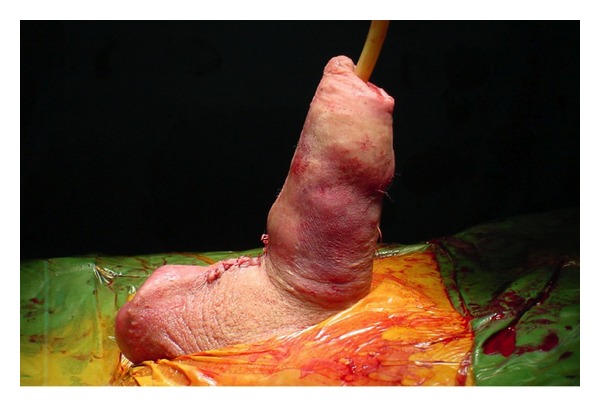
After having adjusted the tension of the second row of plication sutures, complete penile straightness with a fully inflated penile prosthesis is seen.

Following spinal anaesthesia, the genital area was prepared. Artificial erection with ICI of saline and occlusion at the penile base with finger pressure showed approximately 70° curvature of dorsal penile shaft (Figure 1). A 4 cm midline penoscrotal incision was then carried out and Buck's fascia open for adequate exposure of ventral tunica albuginea bilaterally. Two paired rows of 2/0 nonabsorbable braided polyester sutures were placed at the site of maximal penile curvature [10] but left untied. Corporotomies were made proximal to plication sutures and standard placement of 2-piece inflatable Mark 2 penile prosthesis (Mentor Corp., Santa Barbara, California) was carried out. The prosthesis was inflated and the residual curvature assessed. The first row of plication sutures was tied (Figure 2); tension on the second plication sutures was adjusted till complete penile straightness (Figure 3). We placed no drainage but a light pressure dressing that was removed on postoperative day 1 together with the Foley catheter.

Postoperative course was uneventful and the patient was discharged on postoperative day 2. The patient was scheduled for our standard 6-week training period to learn correct penile prosthesis functioning. At 3-month follow-up, he reported that having resumed satisfactory intercourse, inflation of the prosthesis showed straight penis and IIEF-5 score was 24. To date, 8 years after surgery, he reports normal voiding function, successful intercourse, and straight penis, IIEF-5 score 24. His PSA is 0.002 ng/mL.

## 3. Discussion

Surgical correction of severe penile curvature and erectile dysfunction is a long-standing surgical challenge. Mild-to-moderate penile curvature can often be corrected by cylinder insertion, and the prosthetic material seems to play a role in this respect. Montague et al. [11] reported on the ability of AMS CX and Ultrex cylinders to straighten the penis in men who received the AMS-700 inflatable penile prosthesis (American Medical Systems, Minnetonka, Minnesota); all 34 patients who received CX cylinders achieved complete penile straightening, whereas 10 of 38 who had Ultrex cylinders placed required corporoplasty.

In case of persistent curvature after corporeal dilation and prosthesis placement, Wilson and Delk II [2] suggested “modelling” the penis over the inflated penile prosthesis, a technique that involves manual penile bent opposite to the site of curvature for 90 seconds. In the original study [2], 118 (86%) of 138 patients were able to achieve straight, rigid penis; plaque incision was necessary in 11 (8%). Subsequent long-term comparison of inflatable penile prosthesis implantation with and without penile modelling showed no difference in the revision rate but 4% versus nil urethral laceration rate [3]. Whether an uncontrolled lengthening procedure, such as Wilson's “plaque cracking,” is preferable to standard tunica albuginea lengthening or shortening procedure remains questionable.

Penile lengthening procedures involve incising the short side, with or without patching it. The use of combined penile prosthesis implantation and plaque incision has first been reported by Raz et al. in 1977 [12]. Thereafter, the combination of several types of prostheses with several graft materials [13, 14] and even without grafting [6] has been reported. The experience gained over the years has shown that all of these techniques are associated with an increased risk of penile hematoma, penile numbness due to injury to the neurovascular bundle, graft infection, tunical erosion, and even recurvature due to graft retraction [8].

To our knowledge, there is only one report [8] of combining penile prosthesis implantation with a shortening procedure, namely, tunica albuginea plication. Rahman et al. [8] reported that all five patients they operated on with this procedure had excellent results and no complications at a mean follow-up of 22 months (range 7–36). The main advantages of this technique, which they reserved to patients with severe curvature, included (i) being performed under direct vision, thus avoiding blind manoeuvres that can injure the urethra or the neurovascular bundle; (ii) carrying no risk for hematoma/infection, since there is no tunical opening nor graft placement; and (iii) providing proper penile straightening by adjusting final tension on the plication sutures.

The reported case is the first to provide evidence for long-term efficacy and safety of this technique. Another potential advantage of this technique is being safe for the prosthesis, as plication stitches are placed before prosthesis implantation, whereas the modelling procedure and tunical incision/grafting may both carry some risk of damaging the prosthesis. A potential disadvantage could be penile shortening, but it is difficult to establish whether this is greater than that following patching or modelling procedure, whereby some degree of retraction is always to be expected.

In conclusion, this case provides further evidence for combined penile plication and inflatable penile prosthesis implantation being a simple, safe, and effective means of treating erectile dysfunction and severe penile curvature.
